# Development and internal validation of a nomogram for predicting cognitive impairment after mild ischemic stroke and transient ischemic attack based on cognitive trajectories: a prospective cohort study

**DOI:** 10.3389/fnagi.2025.1427737

**Published:** 2025-01-29

**Authors:** Panpan Zhao, Lin Shi, Guimei Zhang, Chunxiao Wei, Weijie Zhai, Yanxin Shen, Yongchun Wang, Zicheng Wang, Li Sun

**Affiliations:** ^1^Department of Neurology, The First Affiliated Hospital of Henan University, Henan University, Kaifeng, China; ^2^Department of Neurology and Neuroscience Center, The First Hospital of Jilin University, Jilin University, Changchun, China

**Keywords:** cognitive trajectory, ferritin, mild stroke, nomogram, cognitive impairment, prediction model, latent class growth analysis

## Abstract

**Introduction:**

Many predictive models for cognitive impairment after mild stroke and transient ischemic attack are based on cognitive scales at a certain timepoint. We aimed to develop two easy-to-use predictive models based on longitudinal cognitive trajectories to facilitate early identification and treatment.

**Methods:**

This was a prospective cohort study of 556 patients, followed up every 3 months. Patients with at least two cognitive scales within 2.5 years were included in the latent class growth analysis (LCGA). The patients were categorized into two groups based on the LCGA. First, a difference analysis was performed, and further univariate and stepwise backward multifactorial logistic regression was performed. The results were presented as nomograms, and receiver operating characteristic curve analysis, calibration, decision curve analysis, and cross-validation were performed to assess model performance.

**Results:**

The LCGA eventually included 255 patients, and the “22” group was selected for further subgroup analysis. Among them, 29.8% were included in the cognitive impairment trajectory. Model 1, which incorporated baseline Montreal Cognitive Assessment, ferritin, age, and previous stroke, achieved an area under the curve (AUC) of 0.973, and model 2, which incorporated age, previous stroke, education, and ferritin, with an AUC of 0.771. Decision curve analysis and cross-validation showed excellent clinical applicability.

**Discussion:**

Here, we developed two simple and easy-to-use predictive models of post-stroke cognitive trajectories based on a LCGA, which are presented in the form of nomograms suitable for clinical application. These models provide a basis for early detection and prompt treatment.

## 1 Introduction

Post-stroke cognitive impairment (PSCI) is a varying degree of cognitive decline associated with prior definite stroke symptoms caused by any reason (El Husseini et al., 2023). Some patients experience significant acute cognitive decline after a mild stroke and transient ischemic attack (TIA) (Pendlebury and Rothwell, 2019; Hua et al., 2023), with gradual improvement in some patients over several months (Cramer et al., 2023). However, during long-term follow-up, some patients progressively deteriorate (Pendlebury and Rothwell, 2019; El Husseini et al., 2023). Reversible causes of deterioration in cognitive function are a cause for concern. However, there is no consensus on the time frame of PSCI; despite this, 3–6 months is generally used as the cutoff point for diagnosing the disease (Mok et al., 2017). Most current studies analyzed cognitive function at a fixed time, such as 3, 6, and 12 months after stroke, as an outcome variable (Rost et al., 2022), which cannot comprehensively respond to the longitudinal changes in cognitive function after stroke. Therefore, we applied latent class growth analysis (LCGA) to automatically categorize patients into trajectory subgroups for trajectory analysis to provide a more comprehensive and objective depiction of cognitive function trends of patients with stroke.

There is no specific treatment for PSCI. However, many interventions exist, including medications (e.g., cholinesterase inhibitors) and non-pharmacological treatments (e.g., cognitive rehabilitation, transcranial magnetic stimulation, acupuncture, and remote ischemic conditioning) (El Husseini et al., 2023). These tools not only increase the financial burden but may also have certain side effects, making it important to recognize early whether cognitive deficits develop after stroke and TIA. The most widely accepted models are SIGNAL2 (Kandiah et al., 2016) and CHANGE (Chander et al., 2017). The SIGNAL2 model included age, education, acute cortical infarcts, white matter hyperintensity (WMH), chronic lacunae, global cortical atrophy, and intracranial large vessel stenosis, yielding an area under the curve (AUC) of 82.9%, sensitivity and specificity of 82.1% and 68.0% at 3–6 months (respectively), and of 64.7% and 79.17% at 12–18 months (respectively) (Kandiah et al., 2016). The CHANGE predictive model without intracranial stenosis, compared with SIGNAL2, had an AUC of 0.78 at 3–6 months. In addition, a study using a machine-learning predictive model for PSCI indicated that cortical infarcts, medial temporal lobe atrophy, initial stroke extent, stroke history, and strategic infarcts predicted PSCI, with an AUC of 79.19% (Lee et al., 2023). These studies all applied imaging and demographic data and did not include hematological indicators. Therefore, we further developed predictive models based on cognitive trajectories utilizing comprehensive and multivariate demographic, imaging, and clinically relevant data with the aim of early diagnosis and treatment.

## 2 Materials and methods

### 2.1 Study population

**FIGURE 1:**
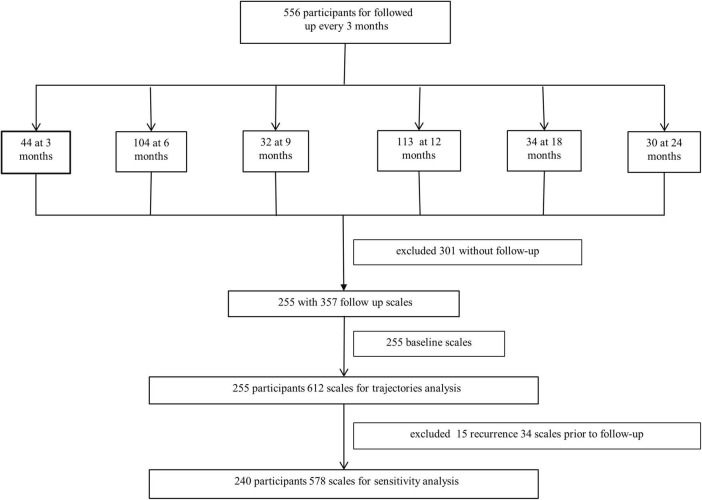
Participant selection flowchart.

This prospective study was conducted at the First Hospital of the Jilin University. It consistently included individuals with mild stroke [National Institutes of Health Stroke Scale (NIHSS) score ≤ 6] and TIA from April 2019 to March 2022. A total of 556 individuals who met the criteria were enrolled and followed up every 3 months for 2.5 years. Two hundred and fifty-five patients with at least two cognitive scales were included in the trajectory analysis. The inclusion criteria were: (1) age 50–80 years, (2) onset of acute cerebral infarction and TIA definitively diagnosed within 2 weeks and NIHSS score ≤ 6, and (3) completed baseline cognitive scales. The exclusion criteria were: (1) presence of other diseases or drug applications affecting cognitive function before stroke, and (2) without follow-up data (Figure 1).

### 2.2 Ethics approval

The research was carried out following the World Medical Association Declaration of Helsinki and was approved by the First Hospital of Jilin University Ethics Committee. The procedures followed conformed to national and institutional guidelines, and written informed consent was obtained from all participants. This study was registered with the Chinese Clinical Trial Registry (https://www.chictr.org.cn/; unique identifier: ChiCTR1900022675).

### 2.3 Cognitive assessment and definition of trajectory timepoints

We performed the first assessment of cognitive and neuropsychiatric function in patients who were relatively stable within 7–10 days after stroke/TIA onset, including the Mini-Mental State Examination (MMSE), Montreal Cognitive Assessment (MoCA). For these assessment scales, we have obtained the copyright license. Subsequent cognitive assessments were performed every 3 months. These scales were completed by full-time evaluators with more than 5 years of experience, with one point added to the MMSE/MoCA score for <12 years of education. We used the MoCA, considered to have good sensitivity and specificity for assessing cognitive function after stroke (Salvadori et al., 2013), as the cognitive outcome. Overall cognitive function after stroke may not stabilize until 12 months after stroke onset (Rost et al., 2022), and therefore we used outcomes obtained every 3 months as a trajectory time point within 1 year, and every 6 months subsequently.

Follow-up data collected at 3–4 months post-stroke was assigned to the 3-month follow-up category, those collected at 5–7 months to the 6-month follow-up category, those collected at 8–10 months to the 9-month follow-up category, those collected at 11–15 months to the 12-month follow-up category, those collected at 16–21 months to the 18-month follow-up category, and those collected at 22–30 months to the 24-month follow-up category.

### 2.4 Study variables

We collected clinical data related to stroke and cognitive impairment at baseline, including sex, age, education, blood pressure, and risk factors associated with cerebrovascular diseases such as smoking, alcohol consumption, hypertension, diabetes mellitus, cardiac disease, and previous stroke. We also gathered hematological indices such as glucose, lipids, uric acid, homocysteine, ferritin, and other iron metabolism indicators. Brain atrophy was defined as cerebral volume loss due to non-brain injury on brain magnetic resonance imaging (MRI). White matter lesions on fluid-attenuated inversion recovery MRI, number of infarcts on diffusion-weighted MRI, and cervicocerebral arterial stenosis detected by ultrasound or magnetic resonance angiography were also recorded within 72 h of stroke/TIA onset. Smoking and drinking were categorized as current, previous, or never, with previous being more than 1 year of abstinence or occasional smoking/drinking. Hypertension and diabetes mellitus were defined as a previous medical history or meeting the diagnostic criteria during hospitalization. Heart disease and stroke were defined as a preexisting medical history. We used the Trial of Org 10172 in Acute Stroke Treatment (TOAST) as a criterion for stroke etiology classification (Adams et al., 1993), the NIHSS score to assess stroke severity (Kwah and Diong, 2014), and the Fazekas scale score, sum of deep white matter hyperintensity (DWMH), and periventricular hyperintensity (PVH) for white matter hyperintensities (Duering et al., 2023). Cervicocerebral arterial stenosis was defined as >50% stenosis. These definitions are consistent with previously unpublished research; since that study is not yet publicly available, we could not provide citations in this report. These definitions were developed based on our research needs and methodology to ensure consistency and comparability.

### 2.5 Statistical analysis

First, we constructed cognitive trajectories using LCGA based on cognitive scales and trajectory timepoints. Subsequently, we built a predictive model using a trajectory-based risk factor selection. Regarding sample size calculation, based on preliminary calculations, the event rate was 29.8%. Four variables were included in the final model, with the modeling requirement (4 × 10 = 40), at least 40 patients with events and 94 without events should be included. Finally, we performed internal validation of the model. Measurement data were expressed as median and interquartile range, and count data were expressed as frequencies and percentages. Univariate logistic regression analyses were applied for comparisons between trajectory groups, and clinically or statistically significant univariate logistic regression variables were included in a stepwise backward multifactor logistic regression to build a predictive model for different trajectory groups and present the model as a nomogram. We then applied receiver operating characteristic (ROC) curves to evaluate the model’s ability to discriminate patients in the cognitively impaired group, calibration to assess the probability consistency between the model-predicted and patients with actual cognitively impairment, and decision curve analysis (DCA) to quantify the net benefit at different threshold probabilities of cognitive impairment. Finally, we used 10-fold cross-validation for the internal validation of model performance. Relapsed patients were excluded from the sensitivity analysis. All missing values were numeric variables. Variables with more than 10% missing data were deleted, and the rest were imputed using the median. A second sensitivity analysis was conducted after excluding patients with imputed data. All statistical analyses were performed using SPSS version 26 (IBM, Armonk, NY) and STATA15.0 (StataCorp, College Station, TX). A two-sided *P* < 0.05 denotes statistical significance.

## 3 Results

### 3.1 Latent class growth analysis

**FIGURE 2:**
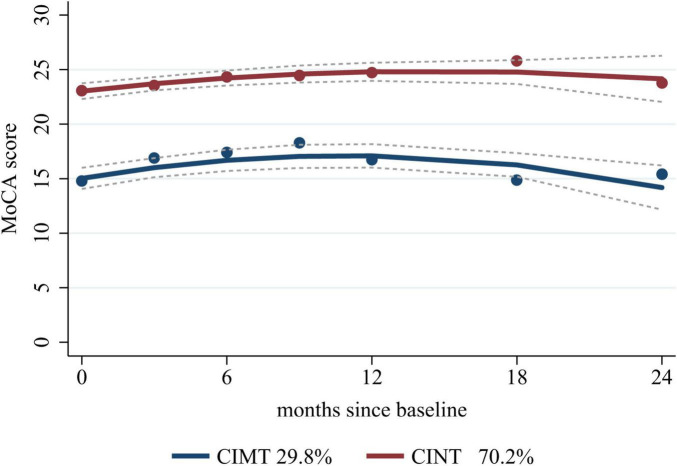
Cognitive trajectories graph. CIMT, cognitive impairment trajectory; CINT, cognitive intact trajectory; MoCA, Montreal Cognitive Assessment.

Based on the eligibility criteria, 255 patients were included in this study. We selected the “22” cognitive trajectories based on the low Akaike information criterion, Bayesian information criterion, high posterior probability (≥0.9), high entropy (>0.8), and >50 cases per trajectory (Mirza et al., 2016; Supplementary Table 1), in which 76 (29.8%) patients were included in the cognitive impairment trajectory (CIMT) and 179 (70.2%) patients were included in the cognitive intact trajectory (CINT). Regardless of baseline values, cognitive function showed a trend of initial improvement followed by gradual decline, reaching the highest value at 12 months after stroke, but the trajectory with cognitive impairment at baseline showed worse cognitive function at 24 months (Figure 2).

### 3.2 Population characteristics

The baseline characteristics are presented in Table 1. Compared with those in the CINT group, patients in the CIMT group were older (median: 63 vs. 60 years), less educated (median: 9 vs. 12 years), and had higher admission systolic blood pressure (SBP, median: 148.5 vs. 142.0 mmHg). While compared with those without previous stroke, patients with previous stroke had a 1.19-fold increased probability of cognitive decline [odds rate (OR) 2.19, 95% confidence interval (CI) 1.21–3.97, *P* = 0.01], and the probability of cognitive decline in patients with brain atrophy was 2.63 times higher (OR 3.63, 95% CI 1.95–6.76, *P* < 0.001). Similar findings were observed in NIHSS score, DWMH, Fazekas score, number of intracranial and cervicocerebral arterial stenoses, and cognitive assessment scales including MMSE and MoCA.

**TABLE 1:** Baseline characteristics of the follow-up patients according to their cognitive trajectory.

Variables	Total	CIMT	CINT	Univariate logistic regression	
	***n* = 255**	***n* = 76**	***n* = 179**	**OR (95% CI)**	***P*-value**
Age	61 (56–68)	63 (59–70.75)	60 (55–67)	1.06 (1.02–1.1)	0.002**
Male	183 (71.8)	50 (65.8)	133 (74.3)	1.5 (0.84–2.69)	0.169
Educational years	12 (9–15)	9 (6–12)	12 (9–15)	0.84 (0.77–0.91)	<0.001***
Systolic pressure (mmHg)	143 (132–159)	148.5 (136.25–165)	142 (130–154)	1.01 (1–1.03)	0.026*
Diastolic pressure (mmHg)	85 (76–94)	87 (78–94.75)	84 (76–93)	1 (0.98–1.02)	0.818
Body mass index (kg/m^2^)	24.51 (22.89–26.9)	24.5 (23.09–26.72)	24.61 (22.76–27.04)	0.99 (0.91–1.08)	0.831
Hypertension (%)	205 (80.4)	66 (86.8)	139 (77.7)	1.9 (0.9–4.03)	0.095
Diabetes (%)	107 (42)	33 (43.4)	74 (41.3)	1.09 (0.63–1.87)	0.758
Heart disease (%)	45 (17.6)	11 (14.5)	34 (19)	0.72 (0.34–1.51)	0.388
Previous stroke (%)	63 (24.7)	27 (35.5)	36 (20.1)	2.19 (1.21–3.97)	0.01**
Smoking				0.96 (0.72–1.27)	0.776
Never (%)	136 (53.3)	43 (56.6)	93 (52)		
Previous (%)	20 (7.8)	3 (3.9)	17 (9.5)		
Now (%)	99 (38.8)	30 (39.5)	69 (38.5)		
Alcohol				0.84 (0.61–1.15)	0.277
Never (%)	148 (58)	51 (67.1)	97 (54.2)		
Previous (%)	40 (15.7)	5 (6.6)	35 (19.6)		
Now (%)	67 (26.3)	20 (26.3)	47 (26.3)		
Diagnose				8.91 (1.17–67.78)	0.035*
TIA (%)	20 (7.8)	1 (1.3)	19 (10.6)		
Cerebral infarction (%)	235 (92.2)	75 (98.7)	160 (89.4)		
Brain atrophy (%)	55 (21.6)	29 (38.2)	26 (14.5)	3.63 (1.95–6.76)	<0.001***
NIHSS score	2 (1–3)	2 (1–3)	2 (1–3)	1.25 (1.04–1.5)	0.016*
PVH score	1 (1–2)	1 (1–2)	1 (1–2)	1.23 (0.86–1.78)	0.258
DWMH score	1 (0–1)	1 (1–2)	1 (0–1)	1.61 (1.15–2.25)	0.006**
Fazekas score	2 (1–3)	2 (2–4)	2 (1–3)	1.22 (1.01–1.47)	0.034*
TOAST				0.83 (0.65–1.06)	0.129
LAA (%)	83 (32.5)	32 (42.1)	51 (28.5)		
Cardioembolism (%)	3 (1.2)	1 (1.3)	2 (1.1)		
Small-vessel occlusion (%)	134 (52.5)	30 (39.5)	104 (58.1)		
Others (%)	35 (13.7)	13 (17.1)	22 (12.3)		
CASN	0 (0–0)	0 (0–0)	0 (0–0)	0.95 (0.64–1.41)	0.802
ICASN	0 (0–1)	0 (0–1)	0 (0–1)	1.52 (1.11–2.07)	0.009**
CCASN	0 (0–1)	0 (0–2)	0 (0–1)	1.21 (0.98–1.51)	0.082
FBG (mmol/L)	5.53 (5.02–6.81)	5.68 (4.98–6.99)	5.5 (5.03–6.69)	1.06 (0.92–1.23)	0.418
Total cholesterol (mmol/L)	4.49 (3.8–5.05)	4.55 (3.91–5.03)	4.41 (3.73–5.11)	1.15 (0.9–1.46)	0.271
Triglyceride (mmol/L)	1.48 (1.11–1.92)	1.64 (1.19–2.03)	1.45 (1.08–1.89)	1.18 (0.96–1.45)	0.124
HDL (mmol/L)	0.98 (0.87–1.16)	1.00 (0.88–1.16)	0.98 (0.87–1.16)	1.11 (0.35–3.49)	0.854
LDL (mmol/L)	2.74 (2.32–3.38)	2.89 (2.40–3.42)	2.69 (2.27–3.34)	1.2 (0.88–1.62)	0.254
Uric acid (μmol/L)	312 (266–372)	288 (259.75–344.5)	317 (270–382)	1 (0.99–1)	0.04*
Homocysteine (μmol/L)	11.9 (10–15.7)	13.09 (10.1–16.73)	11.7 (9.87–15.2)	1.02 (1–1.04)	0.138
Neutrophils (109/L)	4.31 (3.34–5.49)	4.3 (3.33–5.62)	4.33 (3.34–5.34)	1.14 (1–1.29)	0.045*
Lymphocytes (109/L)	1.74 (1.41–2.23)	1.68 (1.33–2.14)	1.79 (1.44–2.26)	0.8 (0.53–1.2)	0.278
Monocytes (109/L)	0.45 (0.35–0.59)	0.47 (0.35–0.63)	0.45 (0.35–0.58)	2.23 (0.67–7.41)	0.191
Fe (μmol/L)	14.6 (11.9–18.1)	14.6 (11.38–16.65)	14.9 (12.2–19.3)	0.97 (0.92–1.02)	0.237
Ferritin (μg/L)	194.9 (130.8–274.8)	208.6 (155.93–313.35)	172 (120.8–255.4)	1 (1–1)	0.021*
TIBC (μmol/L)	47 (42.5–51)	46.7 (41.98–49)	47 (42.5–52.5)	0.96 (0.92–1)	0.061
MMSE	27 (24–29)	22 (19.25–24.75)	28 (26–29)	0.55 (0.47–0.63)	<0.001***
MoCA	21 (17–25)	15 (12–17)	23 (21–26)	0.51 (0.42–0.61)	<0.001***

Numeric variables are presented as median and interquartile range, and count data were expressed as frequencies and percentages (*P < 0.05, **P < 0.01, ***P < 0.001). CASN, carotid arterial stenosis number; CCASN, cervicocerebral arterial stenosis number; CIMT, cognitive impairment trajectory; CINT, cognitive intact trajectory; DWMH, deep white matter hyperintensities; FBG, fasting blood glucose; HDL, high-density lipoprotein; ICASN, intracranial arterial stenosis number; LAA, large-artery atherosclerosis; LDL, low-density lipoprotein; LMR, lymphocyte-to-monocyte ratio; MMSE, Mini-Mental State Examination; MoCA, Montreal Cognitive Assessment; NIHSS, National Institutes of Health Stroke Scale; TIA, transient ischemic attack; TIBC, total iron binding capacity; TOAST, Trial of Org 10172 in Acute Stroke Treatment.

### 3.3 Nomogram development and validation

After careful consideration, we included indicators that are considered to potentially affect cognition in clinical practice and previous studies, including sex (Exalto et al., 2023) and number of infarcts (Ding et al., 2019), along with variables that showed statistical significance in the univariate logistic regression, into a multifactorial regression for further analysis. First, we included all the above indicators in model 1, and model 2 was built by excluding the cognitive mental scales (Table 2). The final model 1 included age, ferritin, previous stroke, and MoCA with the following equation:


Logit⁢(p)=(0.1711434×[age])+(0.0068815×[ferritin])+

 (1.577801×[previous⁢_⁢stroke])-(0.8110665×[MoCA])+

1.603149

**TABLE 2:** Models based on cognitive trajectories.

	Model 1	Model 2
AUC (95% CI)	0.973 (0.957–0.988)	0.771 (0.707∼0.834)
	OR (95% CI)	OR (95% CI)
Age	1.187 (1.087∼1.295)***	1.086 (1.04∼1.134)***
Previous stroke	4.844 (1.58∼14.856)**	3.728 (1.852∼7.502)***
Ferritin	1.007 (1.002∼1.012)**	1.004 (1.001∼1.006)**
Educational years	–	0.801 (0.731∼0.877)***
MOCA	0.444 (0.349∼0.565)***	–

AUC, area under the curve; CI, confidence interval; MoCA, Montreal Cognitive Assessment; OR, odds ratio.

**P < 0.01,

***P < 0.001.

whereas model 2 included age, ferritin, previous stroke, and educational years, with the following equation:


 Logit(p)=(0.0038726×[ferritin])+(1.315809×

[previous_stroke])-(0.2224969×[educational_years])+

0.0822759×[age])-4.861658).

**FIGURE 3:**
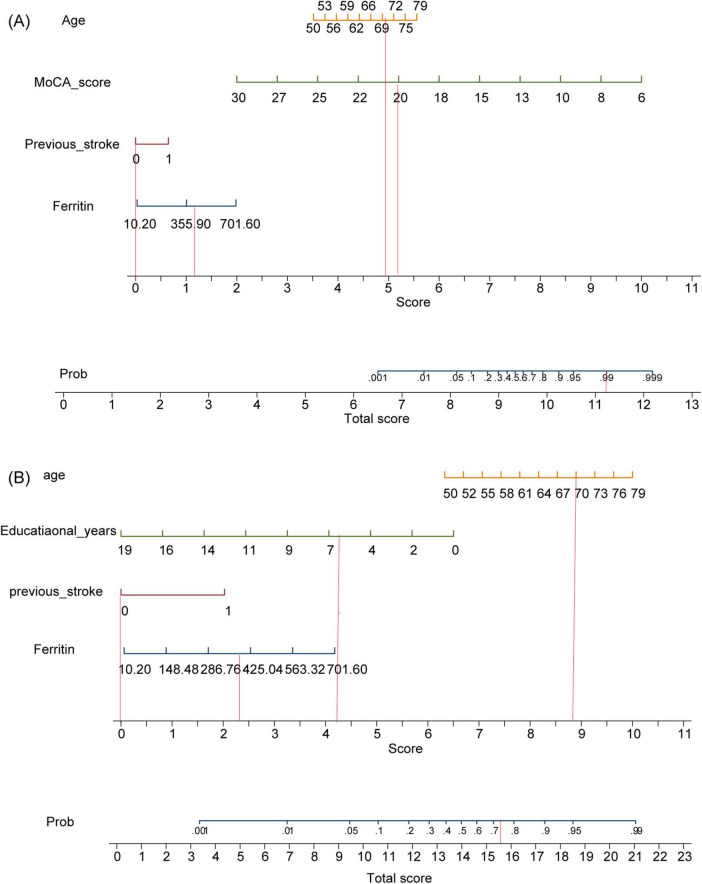
Model nomograms. **(A)** Model 1. **(B)** Model 2. MoCA, Montreal Cognitive Assessment. If a 70-year-old male patient with acute cerebral infarction, who had been educated for 6 years and had no previous history of stroke, was admitted to the hospital with a serum ferritin of 400 ng/ml and a MoCA score of 20, the probability of cognitive impairment in model 1 is 99%, while that in model 2 is 73%.

**FIGURE 4:**
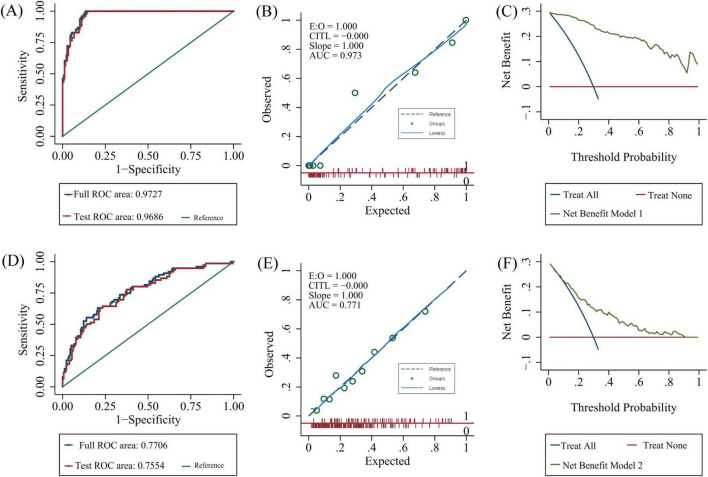
Model validation. Model 1: (**A**) 10-fold cross-validation, **(B)** calibration plots, **(C)** decision curve analysis. Model 2: **(D)** 10-fold cross-validation, **(E)** calibration plots, **(F)** decision curve analysis. AUC, area under the curve; ROC, receiver operating characteristic.

Both models were represented as nomograms (Figure 3). The AUCs were estimated separately to assess the discriminative ability of the above models, yielding values of 0.97 (95% CI 0.96–0.99) for model 1 and 0.77 (95% CI 0.71–0.83) for model 2. Excluding patients with recurrent stroke, the AUC for model 1 reached 0.97 (95% CI 0.96–0.99) and 0.78 (95% CI 0.71–0.84) for model 2 (Supplementary Table 2 and Supplementary Figure 1), sensitivity analysis was also performed by excluding patients with missing data, yielding AUCs of 0.98 (95% CI 0.97–0.99) for model 1 and 0.78 (95% CI 0.72–0.85) for model 2 (Supplementary Table 3 and Supplementary Figure 2). The calibration plots overlapped with the ideal line, indicating that the predicted models sufficiently agreed with the actual observations (Figure 4). The threshold probabilities of standardized net gains in detecting cognitive impairment were determined by applying the nomogram based on DCA. The AUCs corresponding to the 10-fold internal validation were 0.97 and 0.75 for models 1 and 2, respectively.

## 4 Discussion

In this study, we built two cognitive trajectories to predict cognitive function trends after stroke and TIA, in which 29.8% of the patients were included in the CIMT group and 70.2% were included in the CINT group. A slow improvement in cognitive function occurred within the first 12 months after a mild ischemic event, followed by a decline, but patients with poorer cognitive function at baseline had worse cognitive levels at 24 months. Based on the aforementioned cognitive trajectories, we constructed predictive model1 of age, previous stroke, serum ferritin, MoCA with AUC of 0.973 (95% CI 0.957–0.988) and model 2 of age, previous stroke, serum ferritin, years of education with AUC of 0.771 (95% CI 0.707–0.834). Serum ferritin may play a role in the development of PSCI.

We observed an initial improvement followed by deterioration in cognitive function after stroke and TIA based on the two cognitive trajectories. Those with better cognitive function at baseline did not show a significant change at 24 months. However, those with poor cognitive function at baseline had worse cognitive function, in line with previous studies (El Husseini et al., 2023; Lo et al., 2023). This cognitive impairment over time may be because of stroke-induced structural disconnection (Pan et al., 2023), circuit disruption, inflammatory mediators caused by acute ischemia, and related complications (Pendlebury and Rothwell, 2019).

Current studies on cognitive impairment after stroke and TIA have applied inconsistent assessment scales (Lo et al., 2023). The Oxford Vascular Study applied an MMSE < 24 at baseline and follow-up as a diagnostic criterion (Pendlebury and Rothwell, 2019), while the Atherosclerosis Risk in Communities (ARIC) study employed 3- and 10-test batteries (Koton et al., 2022). We used the MoCA as the cognitive outcome, which has high sensitivity and specificity for evaluating cognitive function after stroke (Zietemann et al., 2018; El Husseini et al., 2023). We created two models because the cognitive assessment scales in model 1 were affected by many factors, such as the patient’s medical conditions, and there was a certain degree of subjectivity. The first model included age, serum ferritin level, previous stroke, and MoCA, whereas the second model included age, ferritin level, previous stroke, and educational years. We developed nomograms and internally validated two models. Both models exhibited good predictive performance and clinical utility, as assessed by their AUCs (0.973 and 0.771, respectively), calibration plots, and DCA. Compared with previously mentioned longitudinal data models, SIGNAL2 and CHANGE, our models incorporated more variables, resulting in two models that are simpler, easier to use, and more applicable in the clinical setting.

Models 1 and 2 both included age and previous stroke, and model 1 was significantly better than model 2, indicating that baseline cognitive function strongly predicted long-term cognitive function trajectory. Consistent with our findings, several large international longitudinal studies have indicated that age, previous stroke, stroke severity, low education, white matter lesions, and baseline cognitive function play important roles in PSCI (Pendlebury and Rothwell, 2019; Koton et al., 2022). Our previous exploration of the relationship between indicators of peripheral inflammation and cognitive deficits after stroke within 3–12 months also found that age, previous stroke, and limited education independently influenced cognitive function. These results further indicate the robustness of the models.

Interestingly, both models screened using multifactor logistic regression included serum ferritin levels. Ferritin is the site of intracellular iron storage in various organisms to avoid oxidative stress caused by an excess of free iron. Ferritin plays an important role in maintaining iron homeostasis (Zandman-Goddard and Shoenfeld, 2007; Zhang et al., 2021; Lee and Hyun, 2023; Shesh and Connor, 2023). Serum ferritin is a biomarker of iron loading of the organism (Ayton et al., 2023). Excessive iron loading in the body causes various diseases via Fenton’s reaction. As more studies emerge, ferritin has been brought back into the spotlight. Along with being an iron storage protein and an acute-phase response protein that reacts to the inflammatory and autoimmune state of the body (Zhang et al., 2021; Mahroum et al., 2022), ferritin is also involved in Alzheimer’s disease and neurodegenerative diseases in a different way (Sternberg et al., 2017; Ayton et al., 2023; Lee and Hyun, 2023). A clinical study demonstrated that cognitive dysfunction after cerebral hemorrhage is associated with elevated serum ferritin (Lan et al., 2021). Several clinical studies have demonstrated that elevated serum ferritin levels are associated with a higher risk of ischemic stroke (Ru et al., 2024) and stroke severity (Dávalos et al., 2000; Ru et al., 2024). The degree of stroke severity is strongly associated with PSCI (Koton et al., 2022). However, studies on serum ferritin and cognitive impairment after TIA and acute ischemic stroke are scarce.

Previous studies have used certain timepoints, e.g., 3–6 months (Kandiah et al., 2016; Chander et al., 2017) and 6–12 months (Dong et al., 2021; Gong et al., 2021) and thresholds, e.g., MoCA scores below 22 points (Wei et al., 2023) or 26 points (Zietemann et al., 2018) as endpoints. We applied all the available scale data from the follow-up patients, which can more comprehensively and objectively respond to cognitive changes. More indicators were included, which can reflect the real state of the patients as much as possible and respond to the true situation of the patients. Furthermore, the models were still excellent after excluding patients who had recurrence, which demonstrated their robustness.

We are the first to establish predictive models for cognitive impairment based on cognitive trajectories. However, our study has some limitations. First, this was a single-center study, and a large proportion of patients were lost during follow-up due to the COVID-19 pandemic and other reasons. We found that patients in the follow-up group had relatively better cognitive function, higher levels of literacy, a lower number of arterial stenoses in the head and neck, less severe WMH, and lower systolic blood pressure and serum homocysteine at admission compared to patients lost to follow-up (Supplementary Table 4). Second, we did not perform a systematic assessment of cognitive function prior to the ischemic event, although a detailed medical history was taken to rule out a possible prior cognitive impairment; in addition, because of the limited number of cases, we did not analyze patients with TIA and those with cerebral infarctions separately. Third, PSCI may be affected by stroke treatment methods and methods to improve intelligence, such as medication and cognitive rehabilitation. Many factors affect serum ferritin, including tumors and inflammation (Shesh and Connor, 2023), and we may not have adjusted for all of them. Serum ferritin was not followed up in parallel with cognition scales, which did not allow us to study the different roles played by serum ferritin at different times after stroke. Finally, because of the small number of endpoint variables, we did not categorize the patients into a training and a validation set and did not carry out an external validation. However, we applied internal cross-validation and sensitivity analyses, and found the model to be stable. Additional studies are needed to validate our results.

Overall, we constructed two predictive models for cognitive impairment after mild ischemic stroke and TIA based on cognitive trajectories, and found that baseline cognitive assessments were important, and baseline serum ferritin were independent risk factors affecting cognitive trajectory. Further validation can be conducted in relevant clinical studies, and interventions targeting the reversible risk factor ferritin can be carried out in related basic research to assist in the early detection and timely treatment of PSCI.

## Data Availability

The raw data supporting the conclusions of this article will be made available by the authors, without undue reservation.
