# Supplementary material for: Development and internal validation of a nomogram for predicting cognitive impairment after mild ischemic stroke and transient ischemic attack based on cognitive trajectories: a prospective cohort study
Source: Front Aging Neurosci. 2025 Jan 29;17:1427737. doi: 10.3389/fnagi.2025.1427737 (PMC11814159; doi:10.3389/fnagi.2025.1427737)
Supplement: Supplementary file 1 [file Data_Sheet_1.docx]

Supplementary Material

# Supplementary Figures and Tables

## Supplementary Figures

**Figure S1**：sensitivity analysis by excluding relapsed patients.


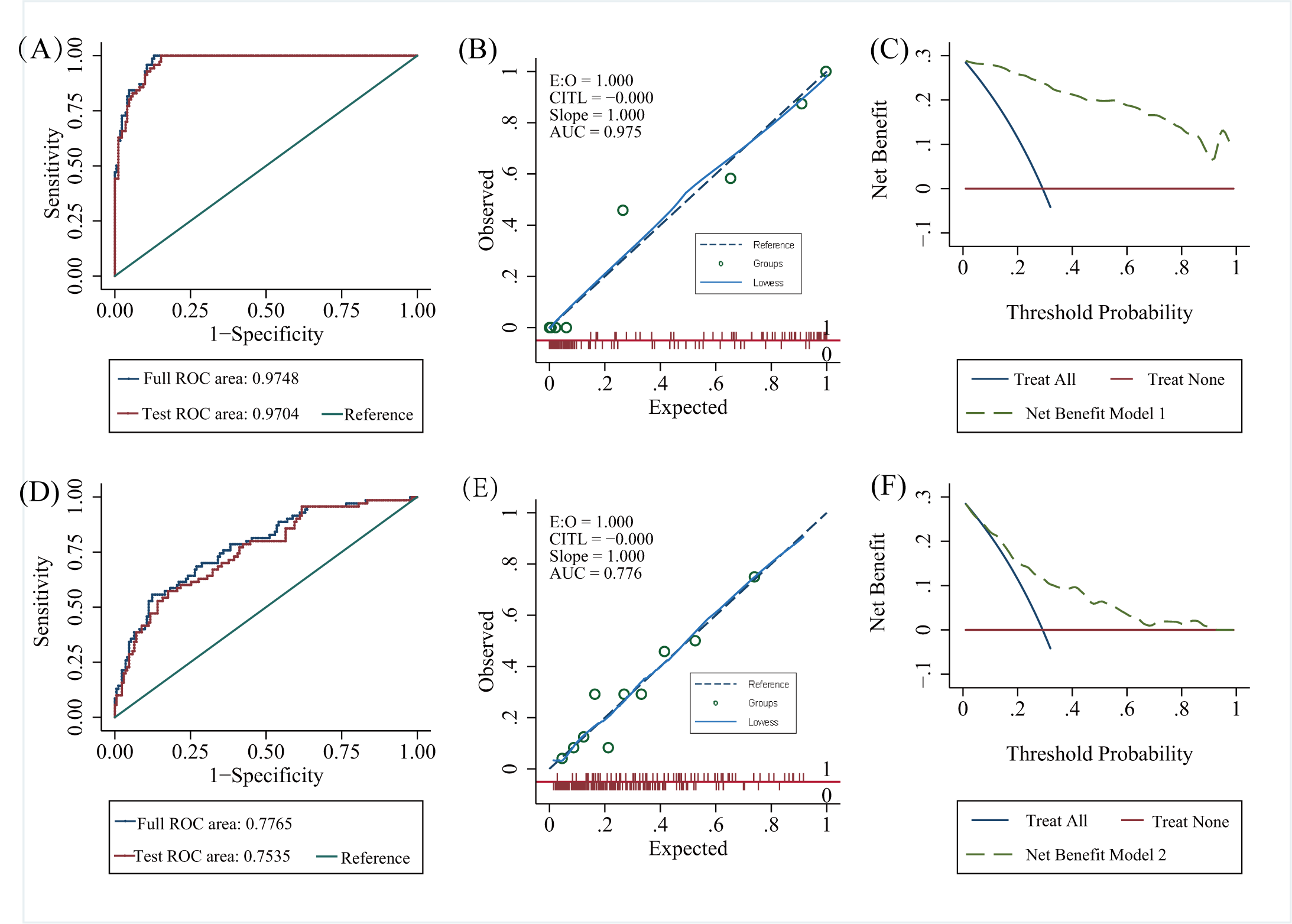


1. Model 1, 10-fold cross-validation, (B) Model 1, The calibration plots, (C) Model 1, Decision curve analysis; (D) Model 2, 10-fold cross-validation, (E) Model 2, The calibration plots, (F) Model 2, Decision curve analysis. Abbreviations: AUC, area under the curve; ROC, receiver operating characteristic.

**Figure S2**：sensitivity analysis by excluding patients with imputed data.


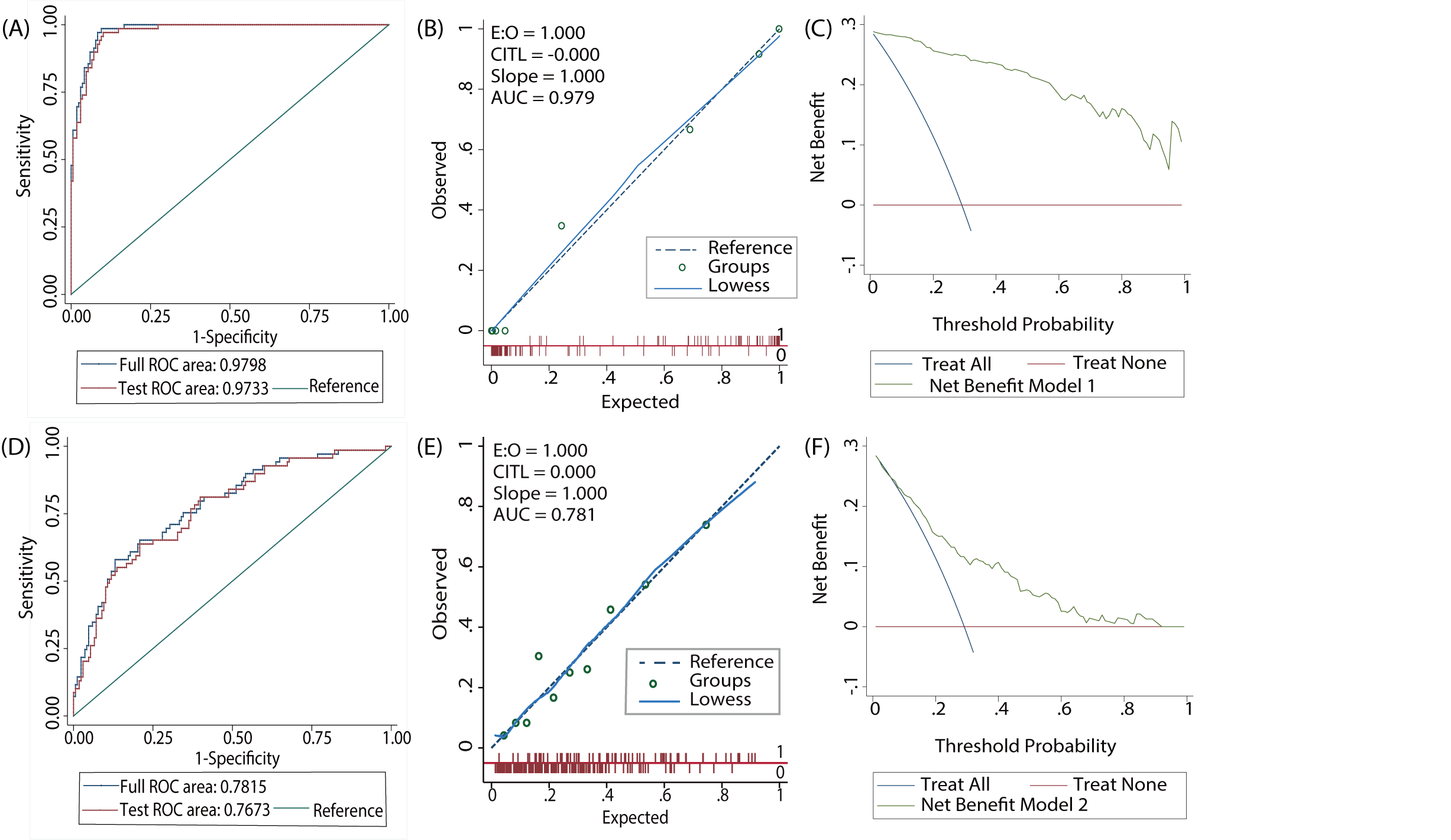


1. Model 1, 10-fold cross-validation, (B) Model 1, The calibration plots, (C) Model 1, Decision curve analysis; (D) Model 2, 10-fold cross-validation, (E) Model 2, The calibration plots, (F) Model 2, Decision curve analysis. Abbreviations: AUC, area under the curve; ROC, receiver operating characteristic.

## Supplementary Tables

**Table S1**：The latent class growth analysis

| LCGA | posterior probability | | BIC | | entropy |
| --- | --- | --- | --- | --- | --- |
|  | Group1 | Group2 | n= 612 | n= 255 |  |
| 22 | 0.918 | 0.947 | -1763.19 | -1759.69 | 0.805 |
| 23 | 0.923 | 0.946 | -1765.98 | -1762.04 | 0.8 |
| 32 | 0.919 | 0.944 | -1761.79 | -1757.85 | 0.8 |
| 33 | 0.912 | 0.947 | -1764.76 | -1760.38 | 0.8 |

Abbreviations: BIC, Bayesian information criterion; LCGA, latent class growth analysis.

**Table S2：**Models based on cognitive trajectories by excluding relapsed patients.

|  | Model 1 | Model 2 |
| --- | --- | --- |
| AUC(95%CI) | 0.975(0.96-0.99) | 0.777(0.712-0.841) |
|  | OR(95%CI) | OR(95%CI) |
| Age | 1.196(1.09-1.313)*** | 1.092(1.045-1.143)*** |
| previous stroke | 5.438(1.632-18.121)** | 3.996(1.922-8.31)*** |
| Ferritin | 1.007(1.002-1.012)** | 1.004(1.001-1.006)** |
| Educational years | --- | 0.8(0.727-0.88)*** |
| MOCA | 0.437(0.338-0.565)*** | --- |

**Table S3：**Models based on cognitive trajectories by excluding patients with imputed data.

|  | Model 1 | Model 2 |
| --- | --- | --- |
| AUC(95%CI) | 0.979(0.966-0.993) | 0.782(0.717-0.846) |
|  | OR(95%CI) | OR(95%CI) |
| Age | 1.209(1.094-1.336)*** | 1.091(1.042-1.141)*** |
| previous stroke | 3.615(1.074-12.163)* | 4.007(1.917-8.377)*** |
| Ferritin | 1.008(1.003-1.013)** | 1.004(1.002-1.007)** |
| Educational years | --- | 0.792(0.719-0.873)*** |
| MOCA | 0.389(0.286-0.529)*** | --- |

**Table S4：**Baseline characteristics between followed-up and non-followed-up patients.

| Variables | Total | with follow-up | without follow-up | *P* |
| --- | --- | --- | --- | --- |
|  | n=556 | n=255 | n=301 |  |
| Age | 62(56~68) | 61(56-68) | 62(57~69) | 0.1 |
| male | 398(71.6) | 183(71.8) | 215(71.4) | 0.93 |
| Educational years | 9(9~12) | 12(9-15) | 9(7.5~12) | <0.001*** |
| Systolic pressure (mmHg) | 147(135~164) | 143(132-159) | 152(137~166.5) | <0.001*** |
| Diastolic pressure (mmHg) | 86(78~95) | 85(76-94) | 87(78~96) | 0.094 |
| Body mass index (kg/m2) | 24.51(23.03~26.7) | 24.51(22.89-26.9) | 24.51(23.15~26.57) | 0.888 |
| Hypertension (%) | 457(82.2) | 205(80.4) | 252(83.7) | 0.307 |
| Diabetes (%) | 254(45.7) | 107(42) | 147(48.8) | 0.105 |
| Heart disease (%) | 91(16.4) | 45(17.6) | 46(15.3) | 0.453 |
| Previous stroke (%) | 150(27) | 63(24.7) | 87(28.9) | 0.266 |
| Smoking |  |  |  | 0.248 |
| Never (%) | 281(50.5) | 136(53.3) | 145(48.2) |  |
| Previous (%) | 55(9.9) | 20(7.8) | 35(11.6) |  |
| Now (%) | 220(39.6) | 99(38.8) | 121(40.2) |  |
| Alcohol |  |  |  | 0.952 |
| Never (%) | 322(57.9) | 148(58) | 174(57.8) |  |
| Previous (%) | 85(15.3) | 40(15.7) | 45(15) |  |
| Now (%) | 149(26.8) | 67(26.3) | 82(27.2) |  |
| Diagnose |  |  |  | 0.812 |
| TIA(%) | 42(7.6) | 20(7.8) | 22(7.3) |  |
| Cerebral infarction (%) | 514(92.4) | 235(92.2) | 279(92.7) |  |
| Brain atrophy (%) | 135(24.3) | 55(21.6) | 80(26.6) | 0.17 |
| NIHSS score | 2(1~3) | 2(1-3) | 2(1~3) | 0.119 |
| PVH score | 1(1~2) | 1(1-2) | 1(1~2) | 0.003** |
| DWMH score | 1(1~1) | 1(0-1) | 1(1~2) | 0.014* |
| Fezakas score | 2(2~3) | 2(1-3) | 2(2~3) | 0.003** |
| Infarcts number |  |  |  | 0.917 |
| 0 (%) | 56(10.1) | 27(10.6) | 29(9.6) |  |
| 1 (%) | 272(48.9) | 123(48.2) | 149(49.5) |  |
| >=2 (%) | 228(41) | 105(41.2) | 123(40.9) |  |
| TOAST |  |  |  | 0.002** |
| LAA (%) | 227(40.8) | 83(32.5) | 144(47.8) |  |
| Cardioembolism (%) | 6(1.1) | 3(1.2) | 3(1) |  |
| Small-vessel occlusion (%) | 248(44.6) | 134(52.5) | 114(37.9) |  |
| Others (%) | 75(13.5) | 35(13.7) | 40(13.3) |  |
| CASN | 0(0~1) | 0(0-0) | 0(0~1) | <0.001*** |
| ICASN | 1(0~1) | 0(0-1) | 1(0~2) | <0.001*** |
| CCASN | 1(0~2) | 0(0-1) | 2(0.5~3) | <0.001*** |
| FBG (mmol/L) | 5.64(5.05~6.88) | 5.53(5.02-6.81) | 5.7(5.1~7.18) | 0.059 |
| Total cholesterol(mmol/L) | 4.5(3.79~5.19) | 4.49(3.8-5.05) | 4.5(3.78~5.29) | 0.322 |
| Triglyceride (mmol/L) | 1.54(1.15~2) | 1.48(1.11-1.92) | 1.56(1.17~2.1) | 0.114 |
| HDL (mmol/L) | 1.01(0.89~1.16) | 0.98(0.87-1.16) | 1.01(0.9~1.17) | 0.267 |
| LDL (mmol/L) | 2.77(2.29~3.36) | 2.74(2.32-3.38) | 2.8(2.28~3.34) | 0.899 |
| Uric acid (umol/L) | 313(265~367) | 312(266-372) | 313(264~365.5) | 0.685 |
| Homocysteine (umol/L) | 12.7(10.56~16.49) | 11.9(10-15.7) | 13(11.16~17.05) | 0.001*** |
| Glycosylated hemoglobin | 6.1(5.7~7.2) | 6.1(5.6-7) | 6.1(5.7~7.4) | 0.199 |
| Leukocyte (109/L) | 6.99(5.86~8.37) | 6.78(5.79-8.18) | 7.1(5.97~8.48) | 0.064 |
| Neutrophils percentage (%) | 63(57~71) | 0.63(0.56-0.7) | 63(57~71) | 0.672 |
| Lymphocytes percentage (%) | 27(21~33) | 0.28(0.21-0.33) | 27(20.5~33) | 0.691 |
| Monocytes percentage(%) | 7(5~8) | 0.07(0.06-0.08) | 7(5~8) | 0.22 |
| Neutrophils (109/L) | 4.37(3.43~5.63) | 4.31(3.34-5.49) | 4.39(3.55~5.7) | 0.261 |
| Lymphocytes (109/L) | 1.79(1.43~2.28) | 1.74(1.41-2.23) | 1.82(1.44~2.36) | 0.435 |
| Monocytes (109/L) | 0.46(0.35~0.59) | 0.45(0.35-0.59) | 0.47(0.35~0.6) | 0.649 |
| Hemoglobin (g/L) | 146(137~156) | 145(136-155) | 146(137~156) | 0.501 |
| Hematocrit (L/L) | 0.43(0.4~0.45) | 0.426(0.397-0.45) | 0.43(0.4~0.45) | 0.558 |
| Platelets (109/L) | 216(184~251) | 210(186-249) | 219(179~253.5) | 0.513 |
| NLR | 2.35(1.72~3.42) | 2.27(1.73-3.24) | 2.43(1.72~3.48) | 0.535 |
| PLR | 119.79(93.48~152.66) | 119.47(96.76-152.9) | 119.79(91.34~152.18) | 0.389 |
| LMR | 3.94(3~5.21) | 3.95(3.07-5.11) | 3.94(2.97~5.31) | 0.667 |
| SII | 511.93(362.12~751.19) | 494.58(349.92-740.2) | 520.35(372.21~778.12) | 0.555 |
| Fe (umol/L) | 14.6(11.8~17.9) | 14.6(11.9-18.1) | 14.6(11.6~17.65) | 0.26 |
| Ferritin (ug/L) | 194.9(130.98~278.88) | 194.9(130.8-274.8) | 194.9(130.05~286.6) | 0.307 |
| TIBC (umol/L) | 47(43~51) | 47(42.5-51) | 47(43~50.7) | 0.802 |
| Iron saturation | 0.31(0.25~0.38) | 0.31(0.25-0.39) | 0.31(0.25~0.38) | 0.471 |
| MMSE | 26(23~28) | 27(24-29) | 26(22~28) | 0.001*** |
| MoCA | 20(16~24) | 21(17-25) | 20(16~24) | 0.005** |

Numeric variables are presented as median and interquartile range, and count data were expressed as frequencies and percentages (*P < 0.05, **P < 0.01, ***P < 0.001).Abbreviations: CASN, carotid arterial stenosis number; CCASN, cervicocerebral arterial stenosis number; CIMT, cognitive impairment trajectory; CINT, cognitive intact trajectory; DWMH, deep white matter hyperintensities; FBG, fasting blood glucose; HDL, high-density lipoprotein; ICASN, intracranial arterial stenosis number; LAA, large-artery atherosclerosis; LDL, low-density lipoprotein; LMR, lymphocyte -to -monocyte ratio; MMSE, Mini-Mental State Examination; MoCA, Montreal Cognitive Assessment; NIHSS, National Institutes of Health Stroke Scale; NLR, neutrophil-to-lymphocyte ratio; NPI, Neuropsychiatric Inventory; PLR, platelet-to-lymphocyte ratio; PVH, periventricular hyperintensity; SII, Systemic Immune Inflammation Index; TIA, transient ischemic attack; TIBC, total iron binding capacity; TOAST, Trial of Org 10172 in Acute Stroke Treatment.
